# Responses of diazotrophic network structure and community diversity to alfalfa-maize intercropping are soil property-dependent

**DOI:** 10.3389/fmicb.2024.1425898

**Published:** 2024-09-18

**Authors:** Jinglei Zhang, Bo Wu, Guoliang Wang, Jinhong Zhang, Chunlin Jia

**Affiliations:** ^1^Shandong Engineering Research Centre for Ecological Horticultural Plant Breeding, Institute of Leisure Agriculture, Shandong Academy of Agricultural Sciences, Jinan, Shandong, China; ^2^Yellow River Delta Modern Agriculture Research Institute, Shandong Academy of Agricultural Sciences, Dongying, Shandong, China

**Keywords:** intercropping, alfalfa, diazotrophic community, composition, structure, soil properties characteristics

## Abstract

**Introduction:**

Intercropping and soil properties both affect soil diazotrophic communities. However, the specific effects that alfalfa-maize intercropping has on diazotrophic networks and community diversity under different soil properties remain unclear.

**Methods:**

In this study, we investigated the soil diazotrophic communities of two crop systems, alfalfa monoculture (AA) and alfalfa-maize intercropping (A/M), in two sites with similar climates but different soil properties (poor vs. average).

**Results and discussion:**

The diazotrophic network complexity and community diversity were higher at the site with poor soil than at the site with average soil (*p* < 0.05). Community structure also varied significantly between the sites with poor and average soil (*p* < 0.05). This divergence was mainly due to the differences in soil nitrogen, phosphorus, and organic carbon contents between the two sites. At the site with poor soil, the A/M system had lower diazotrophic diversity, lower network complexity and greater competition between diazotrophs than the AA system (*p* < 0.05) because intercropping intensified the soil phosphorus limitation under poor soil conditions. However, in the average soil, it was the A/M system that had an altered diazotrophic structure, with an increased abundance of 11 bacterial genera and a decreased abundance of three bacterial genera (*p* < 0.05).

**Conclusion:**

Our results indicated that the effects of alfalfa-maize intercropping on diazotrophic communities were soil property-dependent.

## 1 Introduction

Crop diversification, including via intercropping, has been recognized as an important strategy for improving or maintaining crop productivity and diversity (Li et al., 2023; Xiao et al., 2023). Alfalfa (*Medicago sativa* L.) and silage maize (*Zea mays* L.) are important forages that have been widely planted in the North China Plain to sustain livestock needed to meet the growing demand for meat and milk (Feng et al., 2022; Zhao et al., 2022; Zhou et al., 2022). Studies have shown that intercropping alfalfa with maize can sustain sufficient forage production while reducing nitrogen (N) inputs because of the biological N fixation (BNF) capacity of alfalfa (Sun et al., 2018; Xu et al., 2021; Nasar et al., 2020; Berti et al., 2021; Xu et al., 2022). Diazotrophs, the main microorganisms involved in BNF, are crucial for crop growth (Rodríguez-Blanco et al., 2015; Han et al., 2019; Xiao et al., 2020a; Fan et al., 2023). However, the effects of alfalfa-maize intercropping on the diazotrophic community are still unclear.

Studies have reported that soil diazotrophs are sensitive to cropping systems (Alleman et al., 2021; Hao et al., 2022). Although many researchers have investigated the effects of legume and non-legume intercropping on the diazotrophic community, the results have varied greatly, with studies reporting an increase (Chen J. et al., 2018), a decrease (Gao et al., 2021) or no change (Solanki et al., 2020) in diversity or abundance. This inconsistency in the response of the diazotrophic community to intercropping may relate to the different soil properties present in each study because diazotrophs have a close relationship with soil physicochemical properties (e.g., soil N, soil organic carbon and pH) (Wang Y. et al., 2017; Yu et al., 2020; Zhu et al., 2022). For example, the soil N content is negatively correlated with diazotrophic diversity in general (Zheng et al., 2023). However, little is known about the response of the diazotrophic community to alfalfa-maize intercropping under different soil properties.

Here, we evaluated the impacts of two crop systems, alfalfa monoculture and alfalfa-maize intercropping, in two sites with similar climates but different soil properties (poor vs. average). We hypothesized that (1) diazotrophic network complexity and community diversity will be higher at the site with poor soil than at the site with average soil; (2) the diazotrophic network and community diversity will respond differently to intercropping at the two sites (poor vs. average soil).

## 2 Materials and methods

### 2.1 Study site and experimental design

This study was conducted in Changyi City (119°4′ E, 37°02′ N) and Yucheng City (118°61′ E, 37°31′ N) of Shandong Province, which have similar climates but differ in their soil properties (Supplementary Figure S1, Table 1). Yucheng has better soil quality (average soil site) than Changyi (poor soil site), including higher soil organic carbon and nutrient contents (Table 1). The soil types of Changyi and Yucheng are salinized fluvo-aquic soil (Liu et al., 2018) and luvo-aquic soil (Jia et al., 2010), respectively, according to the Chinese Soil Taxonomy System. Both Changyi and Yucheng have warm, temperate continental monsoon climates, with annual average temperatures of 12.9°C and 13.1°C, and average precipitations of 589 mm and 593 mm, respectively (Tan et al., 2023; Jia et al., 2010).

**Table 1:** Soil properties of the crop systems at the sites with poor and average soil.

**Soil properties**	**Poor soil site**		**Average soil site**	
	**AA**	**A/M**	**AA**	**A/M**
SOC (g/kg)	6.30 ± 0.58b	6.61 ± 0.45b	13.30 ± 0.58a	13.67 ± 0.46a
TN (g/kg)	0.69 ± 0.07b	0.71 ± 0.06b	1.56 ± 0.06a	1.58 ± 0.06a
TP (g/kg)	0.46 ± 0.02b	0.46 ± 0.02b	0.96 ± 0.04a	1.06 ± 0.05a
AN (mg/kg)	68.24 ± 6.93b	70.35 ± 6.20b	143.19 ± 2.78a	158.82 ± 6.61a
AP (mg/kg)	7.51 ± 0.60b	9.96 ± 1.03b	23.56 ± 2.62a	26.29 ± 3.25a
pH	8.47 ± 0.05a	8.49 ± 0.08a	8.18 ± 0.03b	8.23 ± 0.02b
Salt (g/kg)	1.53 ± 0.17a	0.75 ± 0.17b	0.87 ± 0.18b	0.72 ± 0.18b

Values are mean ± standard error (n = 5). Different lowercase letters indicate a significant difference between AA and A/M or between different sites at p <0.05. AA, alfalfa monoculture; A/M, alfalfa-maize intercropping.

The field experiments involved establishing two crop systems, alfalfa monoculture (AA) and alfalfa-maize intercropping (A/M), simultaneously at the sites with poor and average soil. Alfalfa cultivation was initiated in October 2021. In the third year of alfalfa cultivation, the maize added in to the intercropping system (June 2023) (Supplementary Figure S2). Alfalfa seed was sown at a density of 22.5 kg/ha, and maize was planted at a density of 67,500 plants/ha. The two crop systems were arranged side-by-side, with each crop system covering the same area (0.67 ha). The crop systems were managed in the same way at both sites (Supplementary Table S1).

### 2.2 Soil properties

Soil samples (0–10 cm) were collected using a 3.8 cm diameter soil auger from five subplots of each crop system following the diagonal method, i.e., 5 soil samples per crop system. The soil samples from the sites with poor and average soil were collected on September 16, and September 17, 2023, respectively, during the maize's growing season.

The soil physical and chemical properties were measured according to the methods of Bao (2000). The soil organic carbon (SOC) and total N (TN) were measured using dichromate oxidation and the Kjeldahl method, respectively. Soil total phosphorus (TP) and available phosphorus (AP) were determined using molybdenum-antimony colorimetry and spectrophotometry after sodium bicarbonate extraction, respectively. Soil available nitrogen (AN) was measured by the alkaline diffusion method. Soil pH was assessed using a 1:2.5 ratio of air-dried soil to deionized water. Soil-soluble salt content was determined using the oven-drying method.

### 2.3 Soil diazotrophic community analyses

DNA was extracted from each soil sample using the E.Z.N.A.^®^ soil DNA Kit (Omega Bio-tek, Norcross, GA, USA), following the manufacturer's instructions. The concentration and purity of soil DNA were assessed using a NanoDrop 2000 UV-vis spectrophotometer (Thermo Scientific, Wilmington, USA). The *nif* H gene was amplified using *nif* HF/*nif* HR primers (Rösch and Mergel, 2002), and the PCR products were purified using an AxyPrep DNA Gel Extraction Kit (Axygen Biosciences, Union City, CA, USA). The products were subjected to paired-end sequencing on an Illumina Nextseq 2000 platform (Majorbio Company, Shanghai, China). Raw sequencing reads were quality-filtered and merged by fastp and FLASH, respectively (Chen S. et al., 2018). The sequences were clustered into operational taxonomic units (OTUs) at a 97% similarity level using UPARSE (version 7.1) (Edgar, 2013). Taxonomy assignments for the *nif* H gene were conducted by contrasting the RDP Classifier against the FunGene database (fgr/*nif* H_202012) at a 70% confidence threshold. All samples were rarefied to the minimum sequence count before subsequent analyses. The diversity indices, including richness (Chao 1 index), Shannon-Wiener's diversity and Pielou's evenness of the *nif* H gene were analyzed in mothur on the Majorbio Company platform (www.majorbio.com).

We analyzed the diazotrophic community networks using the “igraph” R package, and only the OTUs with an abundance >0.5% were included in this analysis (De Vries et al., 2018). The Spearman's rank correlations (r > 0.6) and *p*-values (*P* < 0.05) were constructed, and visualized using Gephi (Yu et al., 2023).

### 2.4 Statistical analysis

One-way analysis of variance (one-way ANOVA) was used to assess the differences in soil properties and diazotrophic community attributes (e.g., α-diversity and abundance) between the different crop systems or between the different sites. The χ^2^ test was used to evaluate the proportion of network links between crop systems or between the different sites. Nonmetric multidimensional scaling (NMDS) ordination based on Bray-Curtis distance was used to visualize the diazotrophic community structure (β-diversity), and the difference was tested by permutational multivariate analysis of variance (999 permutations) with the “Adonis” function in the vegan package of R version 4.2.1 (R Core Team, 2018). Random forest (RF) analysis, using the “rfPermute” R package, was used to identify the primary soil characteristics predicting diazotrophic community diversity (α-diversity and β-diversity) across different sites (Jiao et al., 2018). A percent increase in the mean squared error (MSE) of variables represents the importance of the predictor, with higher MSE% values indicating greater importance (Jiao et al., 2018). For each site, the Pearson correlation was employed to assess the relationships between diazotrophic network attributes and community diversity, and soil properties.

## 3 Results

### 3.1 Soil properties of crop systems at different sites

The SOC, TN, TP, AN and AP contents were higher at the site with average soil than at the site with poor soil, but pH and salt were lower at the site with average soil (*p* < 0.05; Table 1).

Compared to AA, the A/M system had little effect on soil physicochemical properties. Only the salt content was lower in the A/M system at the site with poor soil (*p* < 0.05; Table 1).

### 3.2 Diazotrophic community diversity and composition

**Figure 1:**
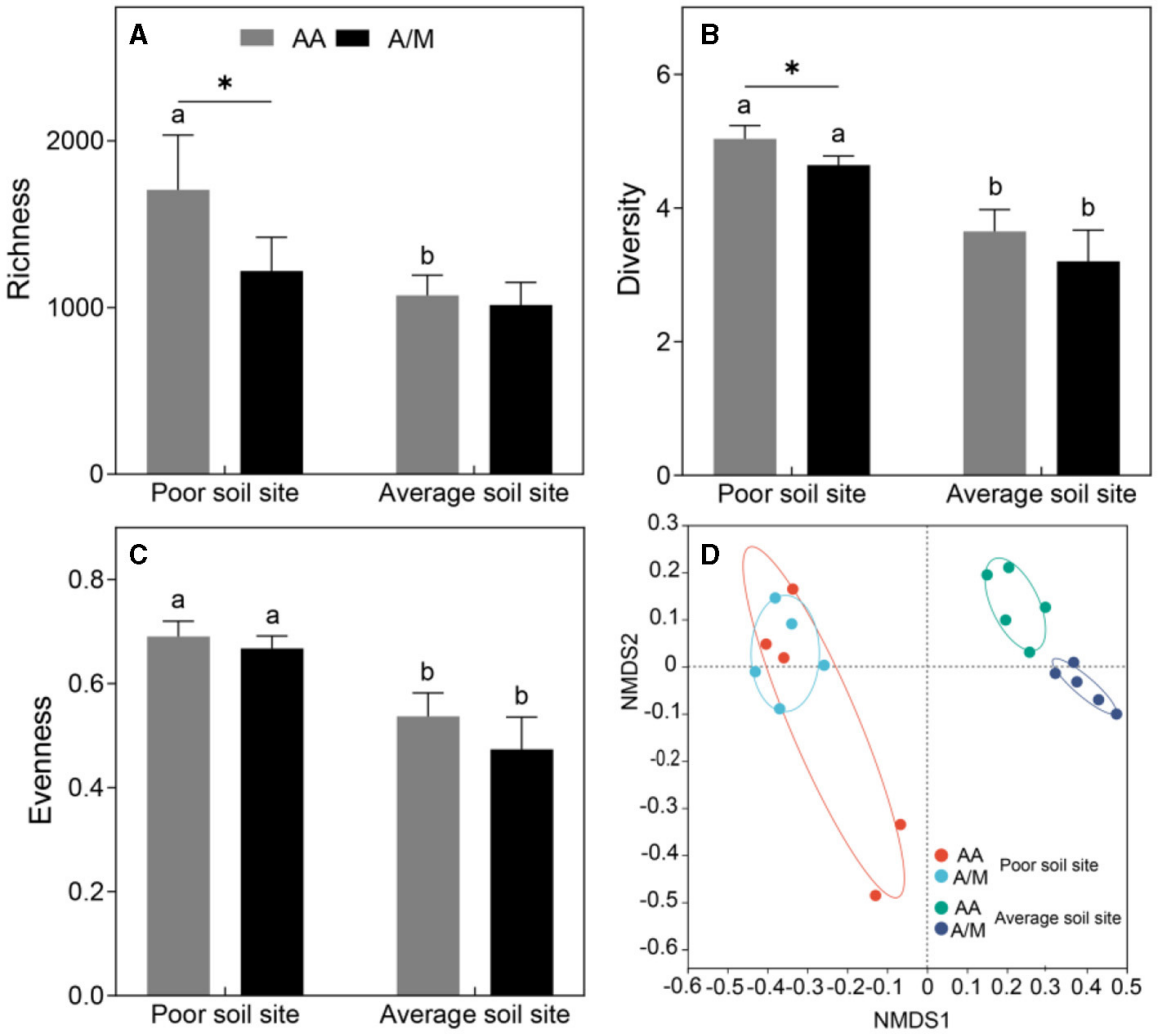
Crop system effects on diazotrophic community diversity **(A–C)** and structure **(D)** at different sites. Lowercase letters and asterisks represent significant (*P* < 0.05) differences between different sites and between crop systems, respectively. AA, alfalfa monoculture; A/M, alfalfa-maize intercropping.

The site with poor soil had a higher diazotrophic community richness than the site with average soil in the AA system only (1705 vs. 1072) (*p* < 0.05; Figure 1A). Diazotrophic diversity was higher at the site with poor soil than at the site with average soil in both the AA system (5.03 vs. 3.65) and the A/M system (4.64 vs. 3.20) (*p* < 0.05; Figure 1B). The site with poor soil had a higher diazotrophic evenness than the site with average soil in both the AA system (5.03 vs. 3.65) and the A/M system (4.64 vs. 3.20) (*p* < 0.05; Figure 1C). The diazotrophic community structure diverged significantly between the two sites (*p* < 0.05; Figure 1D).

The A/M system had significantly lower diazotrophic richness and diversity than the AA system, but only at the site with poor soil (*p* < 0.05; Figures 1A, B). In contrast, the A/M system had a significantly altered diazotrophic community structure, but only at the site with average soil (*p* < 0.05; Figure 1D).

**Figure 2:**
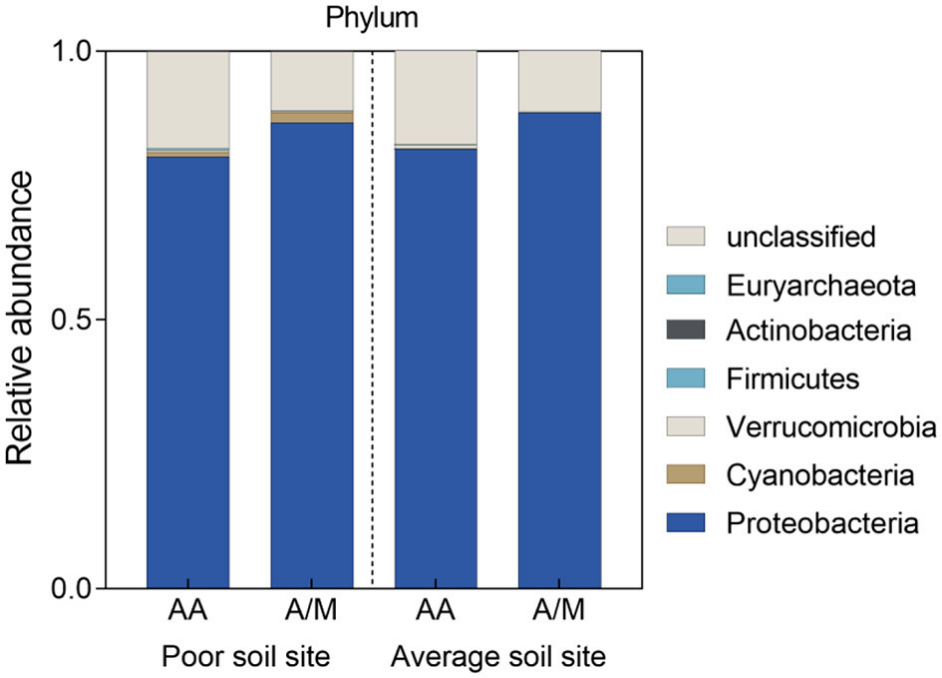
Relative abundance of phyla in the diazotrophic community in two different crop systems at two different sites. AA, alfalfa monoculture; A/M, alfalfa-maize intercropping.

**Figure 3:**
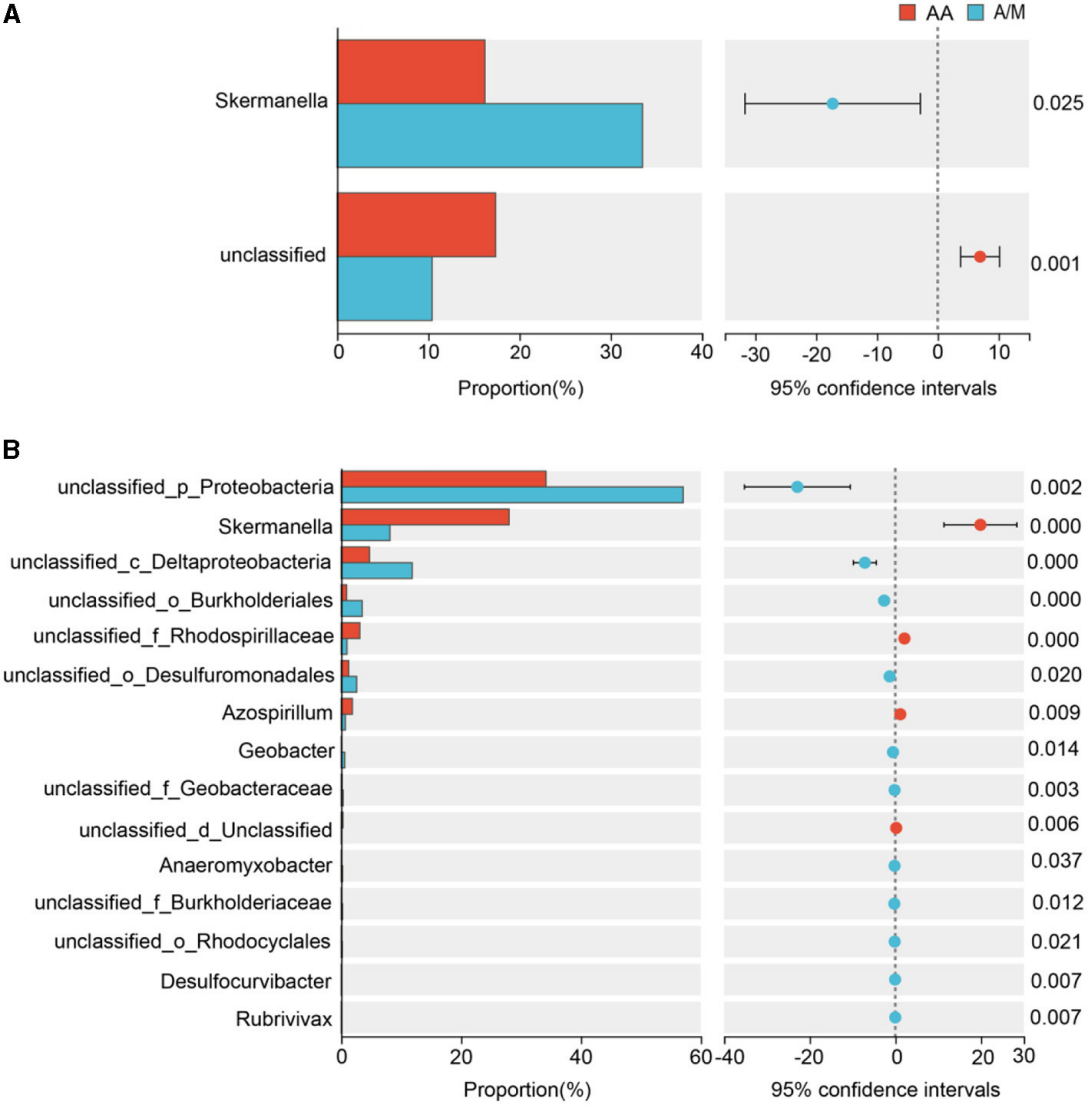
Relative abundance of bacterial genera that responded significantly (*p* < 0.05) to the crop systems at the sites with poor and average soil. AA, alfalfa monoculture; A/M, alfalfa-maize intercropping. **(A)** Poor soil site. **(B)** Average soil site.

Proteobacteria was the dominant phylum across all sites. The A/M system increased the relative abundance of Proteobacteria at the site with poor soil, but it lowered the relative abundance of Cyanobacteria at the site with average soil (*p* < 0.05; Figure 2). A/M increased the relative abundance of *Skermanella* at the site with poor soil (*p* < 0.05; Figure 3). At the site with average soil, A/M increased the relative abundance of 11 bacterial genera, unclassified_p_Proteobacteria, unclassified_c_Deltaproteobacteria, unclassified_o_Burkholderiales, unclassified_o_Desulfuromonadales, *Geobacter*, unclassified_f_Geobacteraceae, *Anaeromyxobacter*, unclassified_f_Burkholderiaceae, unclassified_o_Rhodocyclales, *Desulfocurvibacter*, and *Rubrivivax*, and reduced the relative abundance of three bacterial genera, *Skermanella*, unclassified_f__Rhodospirillaceae and *Azospirillum* (*p* < 0.05; Figure 3).

### 3.3 Diazotrophic community network

**Figure 4:**
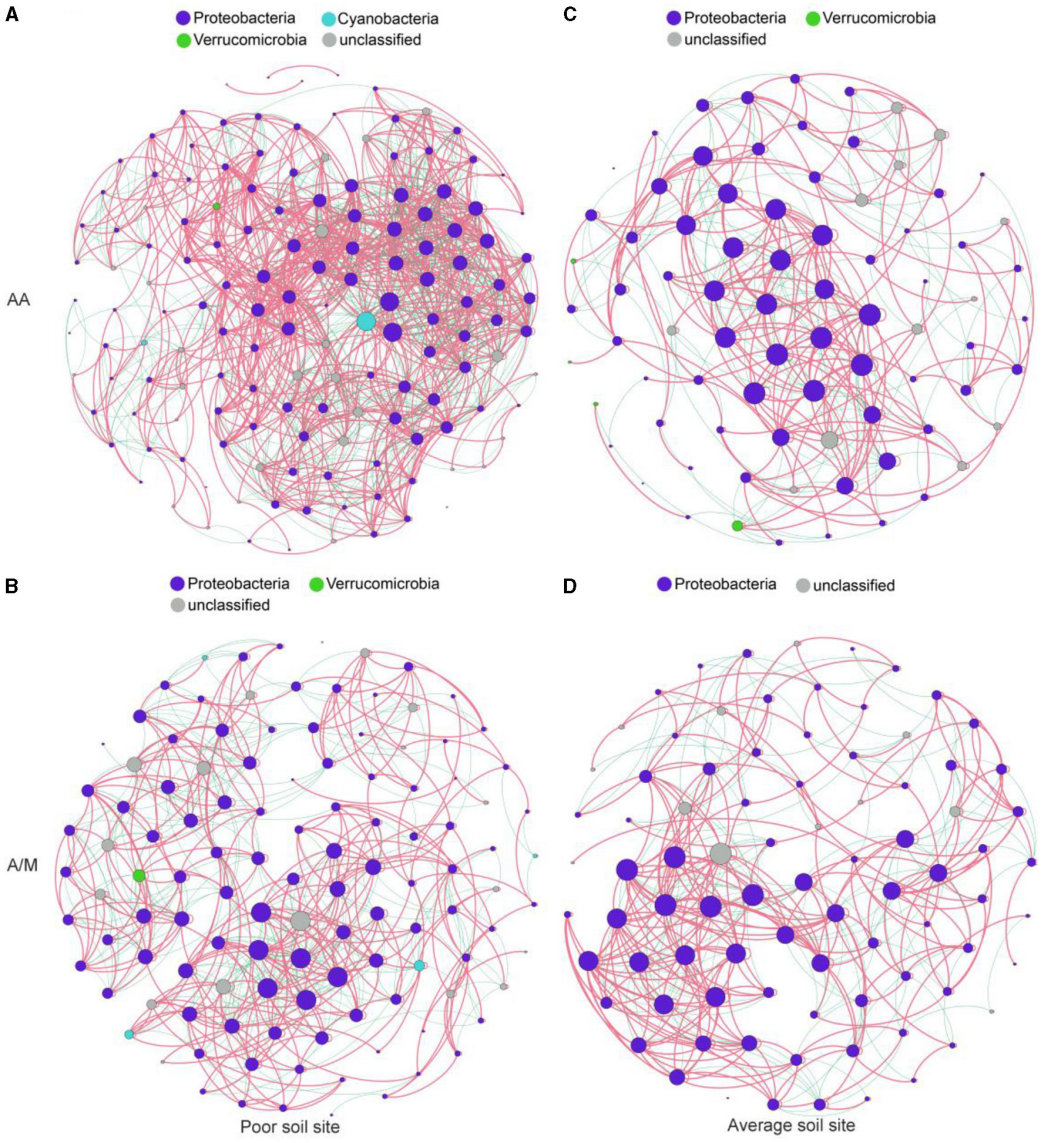
Diazotrophic community networks of AA **(A, C)** and A/M **(B, D)** systems at two different sites. Node size is proportional to the node degree. Red and green edges indicate positive and negative correlations, respectively. AA, alfalfa monoculture; A/M, alfalfa-maize intercropping.

The diazotrophic networks at the site with poor soil had more nodes and links and a higher average degree than those at the site with average soil (Figure 4, Supplementary Table S2). Additionally, the site with poor soil had a higher proportion of negative links than the site with average soil under the A/M system (χ^2^ = 6.50, *p* < 0.05).

Compared to AA, A/M reduced the numbers of network nodes and links and the average degree (*p* < 0.05), but the proportion of negative links increased at the site with poor soil (χ^2^ = 6.50, *p* = 0.07). A/M had no significant effect on the diazotrophic network at the site with average soil (Figure 4, Supplementary Table S2).

### 3.4 Relationships between the diazotrophic community and soil properties

Across study sites, RF analysis showed that AP, pH, and TP were the three most important soil factors for driving diazotrophic richness (Supplementary Figure S3). All soil properties measured affected diazotrophic diversity, except for soil salt (Supplementary Figure S3). Most of the soil properties measured affected diazotrophic evenness, but soil salt and pH did not (Supplementary Figure S3). All soil properties measured affected diazotrophic structure (NMDS1), except for soil salt (Supplementary Figure S3). Most of the soil properties measured affected the diazotrophic network, but AP and pH did not (Supplementary Figure S4).

**Figure 5:**
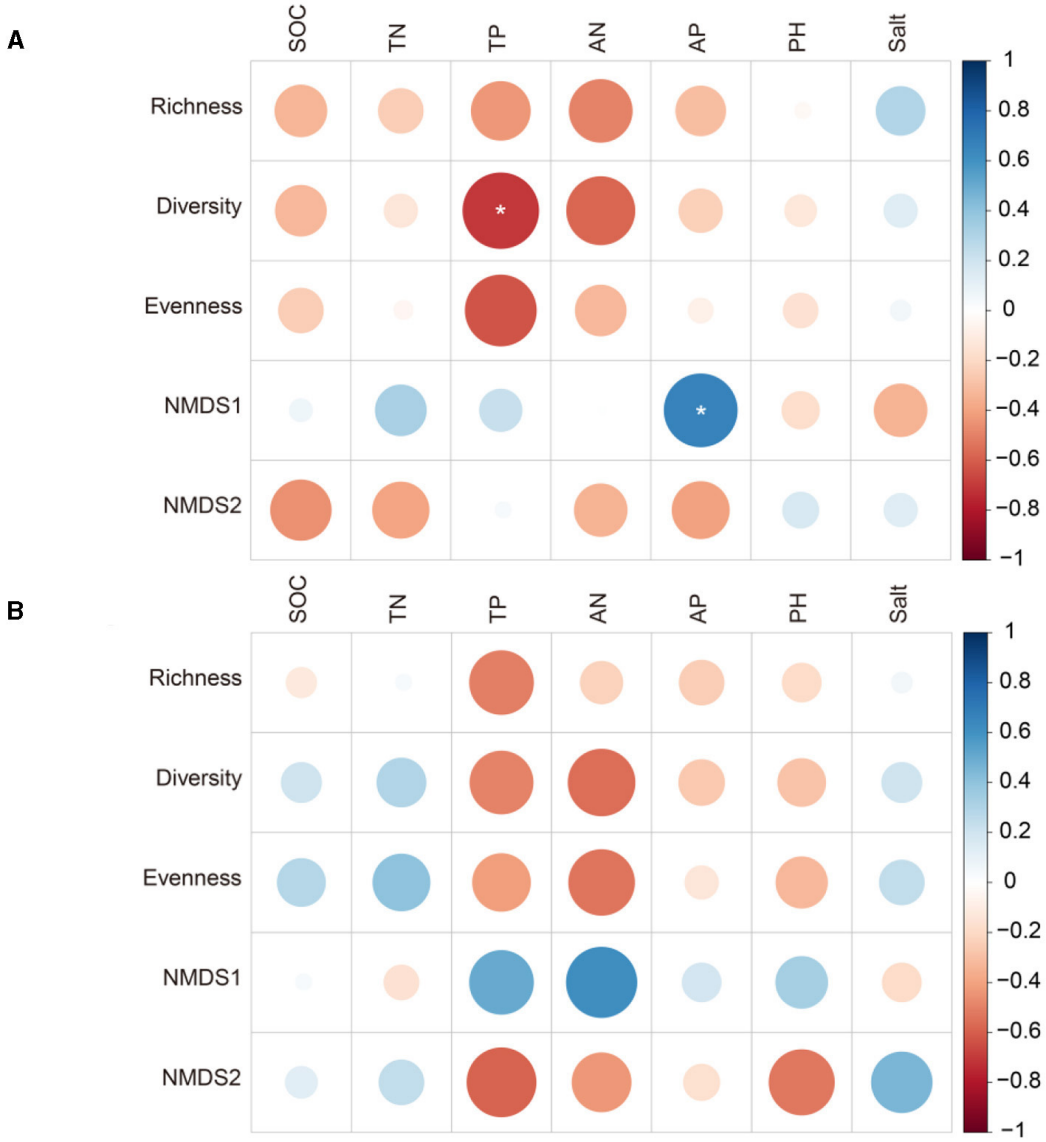
Pearson's correlation (r) between diazotrophic diversity and soil properties at the sites with poor and average soil. ^*^ <0.05. **(A)** Poor soil site. **(B)** Average soil site.

**Figure 6:**
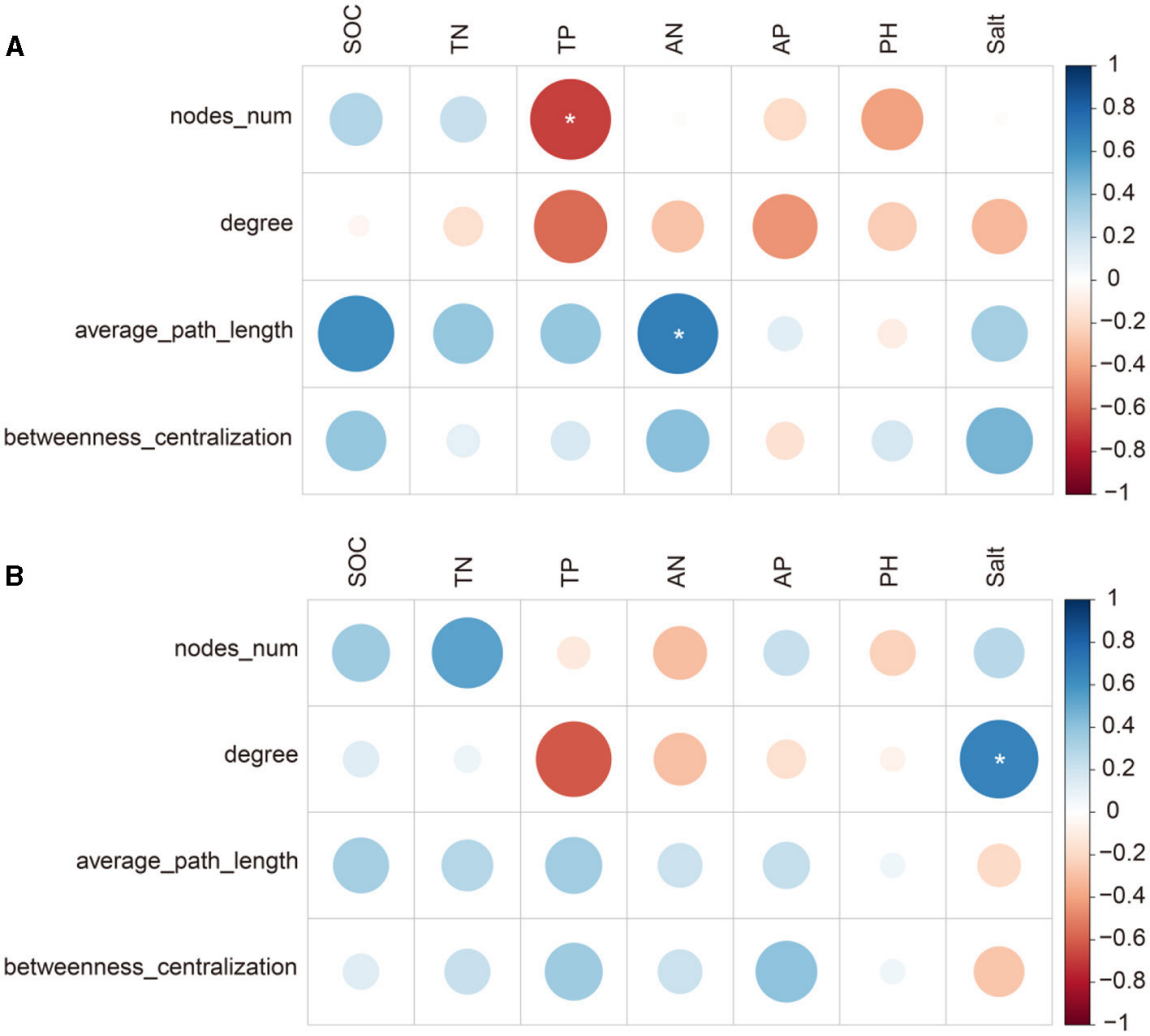
Pearson's correlation (r) between diazotrophic network attributes and soil properties at the sites with poor **(A)** and average soil **(B)**. ^*^ <0.05.

At the site with poor soil, TP related significantly to diazotrophic diversity and the number of network nodes, and AP related significantly to NMDS1 and the average path length of the networks (*p* < 0.05; Figures 5, 6). At the site with poor soil, salt correlated significantly with the network degree (*p* < 0.05; Figure 6).

## 4 Discussion

### 4.1 Responses of diazotrophic diversity to two different crop systems across two different sites

We found that diazotrophic diversity was higher at the site with poor soil than at the site with average soil, mainly because the lower soil N content at the poor site (Supplementary Figure S3), as diazotrophic diversity was negatively correlated with soil N (Zheng et al., 2023). This was consistent with previous studies where long-term N fertilization negatively affected diazotrophic diversity (Wang C. et al., 2017), thereby indicating that high soil N inhibits BNF rate. Studies have further shown that other soil properties, such as soil P, SOC and pH, correlated positively with diazotrophic diversity and also played a key role in driving it (Chen et al., 2021; Han et al., 2019; Wang Y. et al., 2017). We too found that soil P, SOC and pH were important drivers of diazotrophic diversity (Supplementary Figure S3), but pH was the only one that correlated positively with diazotrophic diversity. These inconsistent results may be attributed to the different soil types in the different studies.

Consistent with a previous study (Gao et al., 2021), we found that alfalfa-maize intercropping lowered diazotrophic diversity at the site with poor soil, at least when compared to the alfalfa monoculture, but diversity was not lowered at the site with average soil. Though the variation was statistically insignificant, this may relate to the difference in soil TP between the AA and A/M systems at the site with poor soil (Table 1, Figure 5). Some studies reported that P addition can significantly increase soil diazotrophic diversity (Xiao et al., 2020b). Soil N and P contents were low at the site with poor soil (Table 1). Alfalfa-maize intercropping may promote N fixation via the alfalfa (Yong et al., 2018), but it may also exacerbate P limitation (Solanki et al., 2020), thereby leading to decreased diazotrophic diversity. Some researchers have shown that legume-based systems had higher diazotrophic diversity than do non-legume systems (Yang et al., 2019; do Rego Barros et al., 2021). Thus, the inclusion of maize in an alfalfa system may be accompanied by a decrease in diazotrophic diversity.

### 4.2 Responses of diazotrophic structure to different crop systems across sites

As in a previous study (Pereira et al., 2011), the diazotrophic community structure (e.g. NMDS1) differed between the sites with poor and average soil, independent of the crop systems. This can be mainly attributed to the differences in soil P, N, SOC and pH (Supplementary Figure S3), which were similar to the research of Reardon et al. (2014), who reported that N fertilization had greater effects on diazotrophic structure than did crop type. This indicated that soil properties played a major role in shaping soil diazotrophic community structure.

We found that alfalfa-maize intercropping changed the diazotrophic structure at the site with poor soil, but not at the site with average soil. Studies have shown that plants can recruit specific microbes through root exudates (Zou et al., 2020). Alfalfa and maize secreted different exudates, thereby recruiting different microbial groups that would consequently change the respective diazotrophic community structure. This was further demonstrated by the changes in genus abundance in the intercropping system, where 11 bacterial genera increased in relative abundance and three decreased (Figure 3). Interestingly, intercropping had no significant impact on diazotrophic structure at the site with average soil, which indicated that the effect of intercropping on diazotrophic structure can be regulated to a stronger degree by other factors (e.g., soil properties). However, given that this study covered a short time period, whether long-term intercropping changed diazotrophic structure more strongly deserved investigation in future studies.

### 4.3 Responses of diazotrophic network to different crop systems across sites

We found that the diazotrophic network was more complex at the site with poor soil than at the site with average soil. In the poor soil, the network had more nodes, links and a higher average degree. In general, diazotrophic network was more complex in low-fertility soil than in high-fertility soil (Han et al., 2019). Thus, the more complex network at the site with poor soil may be related to the lower contents of SOC, N and P (Supplementary Figure S4). In addition, we found that, under the A/M system, there was a higher proportion of negative links at the site with poor soil than at the site with average soil. This indicated that the competition for resources among different microorganisms was intensified at the site with poor soil (Coyte et al., 2015).

The results showed that alfalfa-maize intercropping reduced the network complexity, meaning there were fewer nodes and links and a lower average degree, at the site with poor soil only. This may be ascribed to the variation in soil TP between the AA and A/M systems (Figure 6). However, we found that alfalfa-maize intercropping increased the proportion of negative links at the site with poor soil only, thus indicating that intercropping intensified the competition among diazotrophs for limited resources under poor soil conditions (Deng et al., 2016; Yuan et al., 2021). In addition, alfalfa-maize intercropping can create distinctive environmental niches and spatial isolation (Berti et al., 2021), which may lead to even more negative links (Fuhrman, 2009; Berry and Widder, 2014).

## 5 Conclusions

In summary, the diazotrophic community differed significantly between the sites with poor and average soil, with higher community diversity and network complexity found at the site with poor soil. The alfalfa-maize intercropping lowered the diazotrophic community diversity and network complexity, but it increased the competition between diazotrophs, at the site with poor soil. However, at the site with average soil, the intercropping altered the diazotrophic community structure. Our results highlight that the effects of short-term alfalfa-maize intercropping on diazotrophic communities are soil property-dependent, while the effects of long-term intercropping on diazotrophic community will require further study.

## Data Availability

The original contributions presented in the study are included in the article/Supplementary material, further inquiries can be directed to the corresponding author.

## References

[B1] Alleman A. B. Mohammed Y. A. McVay K. A. Khan Q. A. Carr P. Miller J.. (2021). Drivers of diazotroph community structure and co-occurrence in a Northern Great Plains pulse crop rotation system. Appl. Soil Ecol. 157:103737. 10.1016/j.apsoil.2020.103737

[B2] Bao S. D. (2000). Soil Agricultural Chemistry Analysis, 3rd edn. Beijing: China Agriculture Press.

[B3] Berry D. Widder S. (2014). Deciphering microbial interactions and detecting keystone species with co-occurrence networks. Front. Microbiol. 5:219. 10.3389/fmicb.2014.00219 24904535 PMC4033041

[B4] Berti M. T. Cecchin A. Samarappuli D. P. Patel S. Lenssen A. W. Moore K. J.. (2021). Alfalfa established successfully in intercropping with corn in the midwest US. Agronomy 11:1676. 10.3390/agronomy11081676

[B5] Chen H. Zheng C. Qiao Y. Du S. Li W. Zhang X.. (2021). Long-term organic and inorganic fertilization alters the diazotrophic abundance, community structure, and co-occurrence patterns in a vertisol. Sci. Total Environ. 766:142441. 10.1016/j.scitotenv.2020.142441 33097271

[B6] Chen J. Arafat Y. Wu L. Xiao Z. Li Q. Khan M. A.. (2018). Shifts in soil microbial community, soil enzymes and crop yield under peanut/maize intercropping with reduced nitrogen levels. Appl. Soil Ecol. 124, 327–334. 10.1016/j.apsoil.2017.11.010

[B7] Chen S. Zhou Y. Chen Y. Gu J. (2018). fastp: an ultra-fast all-in-one FASTQ preprocessor. Bioinformatics 34, i884–i890. 10.1093/bioinformatics/bty560 30423086 PMC6129281

[B8] Coyte K. Z. Schluter J. Foster K. R. (2015). The ecology of the microbiome: networks, competition, and stability. Science 350, 663–666. 10.1126/science.aad2602 26542567

[B9] De Vries F. T. Griffiths R. I. Bailey M. Craig H. Girlanda M. Gweon H. S.. (2018). Soil bacterial networks are less stable under drought than fungal networks. Nat. Commun. 9:3033. 10.1038/s41467-018-05516-7 30072764 PMC6072794

[B10] Deng Y. Zhang P. Qin Y. Tu Q. Yang Y. He Z.. (2016). Network succession reveals the importance of competition in response to emulsified vegetable oil amendment for uranium bioremediation. Environ. Microbiol. 18, 205–218. 10.1111/1462-2920.12981 26177312

[B11] do Rego Barros F. M. Fracetto F. J. C. Junior M. A. L. Bertini S. C. B. Fracetto G. G. M. (2021). Spatial and seasonal responses of diazotrophs and ammonium-oxidizing bacteria to legume-based silvopastoral systems. Appl. Soil Ecol. 158:103797. 10.1016/j.apsoil.2020.103797

[B12] Edgar R. C. (2013). UPARSE: highly accurate OTU sequences from microbial amplicon reads. Nat. Methods 10, 996–998. 10.1038/nmeth.2604 23955772

[B13] Fan Z. Li R. Guan E. Chen H. Zhao X. Wei G.. (2023). Fertilization regimes affect crop yields through changes of diazotrophic community and gene abundance in soil aggregation. Sci. Total Environ. 866:161359. 10.1016/j.scitotenv.2022.161359 36610631

[B14] Feng Y. Shi Y. Zhao M. Shen H. Xu L. Luo Y.. (2022). Yield and quality properties of alfalfa (*Medicago sativa* L.) and their influencing factors in China. Eur. J. Agron. 141:126637. 10.1016/j.eja.2022.126637 35122623

[B15] Fuhrman J. A. (2009). Microbial community structure and its functional implications. Nature 459, 193–199. 10.1038/nature08058 19444205

[B16] Gao H. Li S. Wu F. (2021). Impact of intercropping on the diazotrophic community in the soils of continuous cucumber cropping systems. Front. Microbiol. 12:630302. 10.3389/fmicb.2021.630302 33868191 PMC8044418

[B17] Han L. L. Wang Q. Shen J. P. Di H. J. Wang J. T. Wei W. X.. (2019). Multiple factors drive the abundance and diversity of the diazotrophic community in typical farmland soils of China. FEMS Microbiol. Ecol. 95:fiz113. 10.1093/femsec/fiz113 31295349

[B18] Hao J. Feng Y. Wang X. Yu Q. Zhang F. Yang G.. (2022). Soil microbial nitrogen-cycling gene abundances in response to crop diversification: a meta-analysis. Sci. Total Environ. 838:156621. 10.1016/j.scitotenv.2022.156621 35691356

[B19] Jia L. Wang W. Li Y. Yang L. (2010). Heavy metals in soil and crops of an intensively farmed area: a case study in Yucheng City, Shandong Province, China. Int. J. Env. Res. Pub. He. 7, 395–412. 10.3390/ijerph7020395 20616981 PMC2872287

[B20] Jiao S. Chen W. Wang J. Du N. Li Q. Wei G. (2018). Soil microbiomes with distinct assemblies through vertical soil profiles drive the cycling of multiple nutrients in reforested ecosystems. Microbiome 6, 1–13. 10.1186/s40168-018-0526-0 30131068 PMC6104017

[B21] Li C. Stomph T. J. Makowski D. Li H. Zhang C. Zhang F.. (2023). The productive performance of intercropping. P. Natl. Acad. Sci. USA. 120:e2201886120. 10.1073/pnas.2201886120 36595678 PMC9926256

[B22] Liu W. Xu X. Lu F. Cao J. Li P. Fu T.. (2018). Three-dimensional mapping of soil salinity in the southern coastal area of Laizhou Bay, China. Land Degrad. Dev. 29, 3772–3782. 10.1002/ldr.3077

[B23] Nasar J. Shao Z. Arshad A. Jones F. G. Liu S. Li C.. (2020). The effect of maize–alfalfa intercropping on the physiological characteristics, nitrogen uptake and yield of maize. Plant Biol. 22, 1140–1149. 10.1111/plb.13157 32609937

[B24] Pereira M. C. Semenov A. V. van Elsas J. D. Salles J. F. (2011). Seasonal variations in the diversity and abundance of diazotrophic communities across soils. FEMS Microbiol. Ecol. 77, 57–68. 10.1111/j.1574-6941.2011.01081.x 21385188

[B25] R Core Team (2018). R: A Language and Environment for Statistical Computing. R Package Version 4.2. 1. Vienna: R Foundation for Statistical Computing.

[B26] Reardon C. L. Gollany H. T. Wuest S. B. (2014). Diazotroph community structure and abundance in wheat–fallow and wheat–pea crop rotations. Soil Biol. Biochem. 69, 406–412. 10.1016/j.soilbio.2013.10.038

[B27] Rodríguez-Blanco A. Sicardi M. Frioni L. (2015). Plant genotype and nitrogen fertilization effects on abundance and diversity of diazotrophic bacteria associated with maize (*Zea mays* L.). Biol. Fert. Soils 51, 391–402. 10.1007/s00374-014-0986-8

[B28] Rösch C. Mergel A. Bothe, H. (2002). Biodiversity of denitrifying and dinitrogen-fixing bacteria in an acid forest soil. Appl. Environ. Microb. 68, 3818–3829. 10.1128/AEM.68.8.3818-3829.2002 12147477 PMC124007

[B29] Solanki M. K. Wang F. Y. Li C. N. Wang Z. Lan T. J. Singh R. K.. (2020). Impact of sugarcane–legume intercropping on diazotrophic microbiome. Sugar Tech. 22, 52–64. 10.1007/s12355-019-00755-4

[B30] Sun T. Li Z. Wu Q. Sheng T. Du M. (2018). Effects of alfalfa intercropping on crop yield, water use efficiency, and overall economic benefit in the corn belt of northeast China. Field Crop Res. 216, 109–119. 10.1016/j.fcr.2017.11.007

[B31] Tan Z. Yuan Y. Gu M. Han Y. Mao L. Tan T.. (2023). Levoglucosan and its isomers in terrestrial sediment as a molecular markers provide direct evidence for the low-temperature fire during the mid-Holocene in the northern Shandong Peninsula of China. Quatern. Int. 661, 22–33. 10.1016/j.quaint.2023.05.010

[B32] Wang C. Zheng M. Song W. Wen S. Wang B. Zhu C.. (2017). Impact of 25 years of inorganic fertilization on diazotrophic abundance and community structure in an acidic soil in southern China. Soil Biol. Biochem. 113, 240–249. 10.1016/j.soilbio.2017.06.019

[B33] Wang Y. Li C. Kou Y. Wang J. Tu B. Li H.. (2017). Soil pH is a major driver of soil diazotrophic community assembly in Qinghai-Tibet alpine meadows. Soil Biol. Biochem. 115, 547–555. 10.1016/j.soilbio.2017.09.024

[B34] Xiao D. Tan Y. Liu X. Yang R. Zhang W. He X.. (2020a). Responses of soil diazotrophs to legume species and density in a karst grassland, southwest China. Agr. Ecosyst. Environ. 288:106707. 10.1016/j.agee.2019.106707

[B35] Xiao D. Xiao L. Che R. Tan Y. Liu X. Yang R.. (2020b). Phosphorus but not nitrogen addition significantly changes diazotroph diversity and community composition in typical karst grassland soil. Agr. Ecosyst. Environ. 301:106987. 10.1016/j.agee.2020.106987

[B36] Xiao X. Han L. Chen H. Wang J. Zhang Y. Hu A. (2023). Intercropping enhances microbial community diversity and ecosystem functioning in maize fields. Front. Microbiol. 13:1084452. 10.3389/fmicb.2022.1084452 36687629 PMC9846038

[B37] Xu R. Zhao H. Liu G. Li Y. Li S. Zhang Y.. (2022). Alfalfa and silage maize intercropping provides comparable productivity and profitability with lower environmental impacts than wheat–maize system in the North China plain. Agr. Syst. 195:103305. 10.1016/j.agsy.2021.103305

[B38] Xu R. X. Zhao H. M. Liu G. B. You Y. L. Ma L. Liu N.. (2021). Effects of nitrogen and maize plant density on forage yield and nitrogen uptake in alfalfa–silage maize relay intercropping system in North China Plain. Field Crop Res. 263:108068. 10.1016/j.fcr.2021.108068

[B39] Yang Y. Feng X. Hu Y. Zeng Z. H. (2019). The diazotrophic community in oat rhizosphere: effects of legume intercropping and crop growth stage. Front. Agric. Sci. Eng. 6, 162–171. 10.15302/J-FASE-2018212

[B40] Yong T. Chen P. Dong Q. Du Q. Yang F. Wang X.. (2018). Optimized nitrogen application methods to improve nitrogen use efficiency and nodule nitrogen fixation in a maize-soybean relay intercropping system. J. Integr. Agr. 17, 664–676. 10.1016/S2095-3119(17)61836-7

[B41] Yu F. Lin J. Xie D. Yao Y. Wang X. Huang Y.. (2020). Soil properties and heavy metal concentrations affect the composition and diversity of the diazotrophs communities associated with different land use types in a mining area. Appl. Soil Ecol. 155:103669. 10.1016/j.apsoil.2020.103669

[B42] Yu T. Nie J. Zang H. Zeng Z. Yang Y. (2023). Peanut-based rotation stabilized diazotrophic communities and increased subsequent wheat yield. Microb. Ecol. 86, 2447–2460. 10.1007/s00248-023-02254-2 37296336

[B43] Yuan M. M. Guo X. Wu L. Zhang Y. Xiao N. Ning D.. (2021). Climate warming enhances microbial network complexity and stability. Nat. Clim. Change 11, 343–348. 10.1038/s41558-021-00989-9

[B44] Zhao M. Feng Y. Shi Y. Shen H. Hu H. Luo Y.. (2022). Yield and quality properties of silage maize and their influencing factors in China. Sci. China Life Sci. 65, 1655–1666. 10.1007/s11427-020-2023-3 35122623

[B45] Zheng M. Xu M. Li D. Deng Q. Mo J. (2023). Negative responses of terrestrial nitrogen fixation to nitrogen addition weaken across increased soil organic carbon levels. Sci. Total Environ. 877:162965. 10.1016/j.scitotenv.2023.162965 36948308

[B46] Zhou Z. Zhang Y. Zhang F. (2022). Community assembly correlates with alfalfa production by mediating rhizosphere soil microbial community composition in different planting years and regimes. Plant Soil 479, 355–370. 10.1007/s11104-022-05525-y

[B47] Zhu C. Friman V. P. Li L. Xu Q. Guo J. Guo S.. (2022). Meta-analysis of diazotrophic signatures across terrestrial ecosystems at the continental scale. Environ. Microbiol. 24, 2013–2028. 10.1111/1462-2920.15984 35362656

[B48] Zou J. Yao Q. Liu J. Li Y. Song F. Liu X.. (2020). Changes of diazotrophic communities in response to cropping systems in a Mollisol of Northeast China. PeerJ. 8, e9550. 10.7717/peerj.9550 32742810 PMC7368428

