# Supplementary material for: Responses of diazotrophic network structure and community diversity to alfalfa-maize intercropping are soil property-dependent
Source: Front Microbiol. 2024 Sep 18;15:1425898. doi: 10.3389/fmicb.2024.1425898 (PMC11445037; doi:10.3389/fmicb.2024.1425898)
Supplement: Supplementary file 1 [file Data_Sheet_1.docx]

**
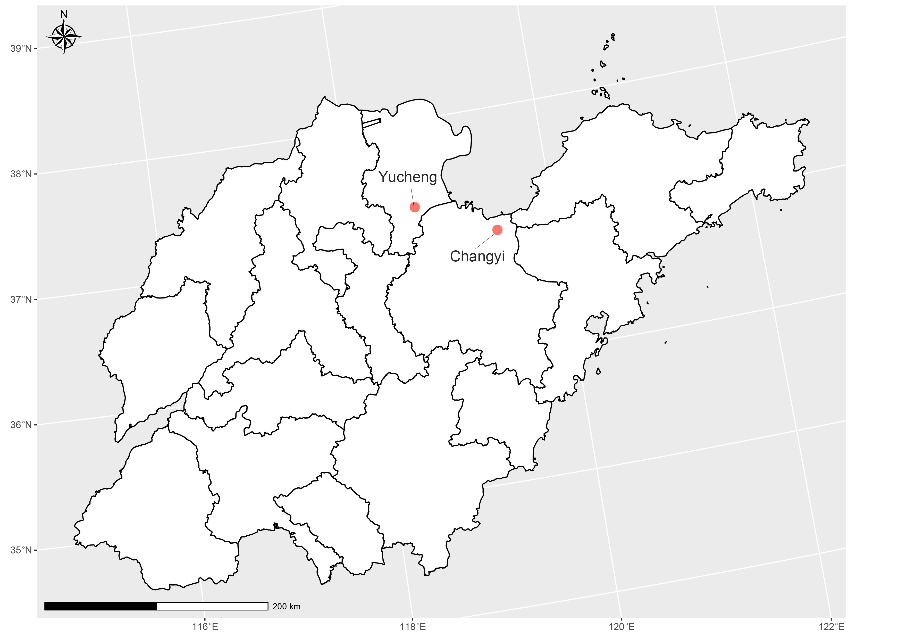
**

**Fig. S1 Location of study sites in Shandong Province**

**
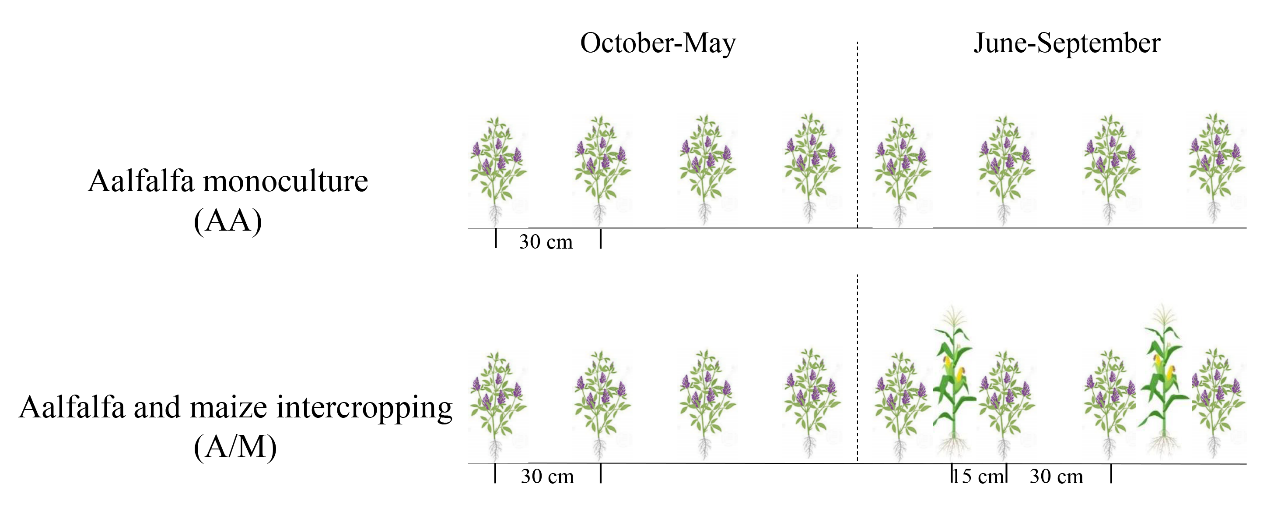
**

**Fig. S2 Schematic diagram of crop systems**

**Table S1 Cropping systems management practices**

| Cropping systems | Basel fertilizer | | Irrigation |
| --- | --- | --- | --- |
| AA | alfalfa (N-P_2_O_5_-K_2_O: 15-42-0, 225 kg/ha^-1^) | | Uniform irrigation according to soil conditions |
| A/M | alfalfa (N-P_2_O_5_-K_2_O: 15-42-0, 225 kg/ha^-1^) | maize (N-P_2_O_5_-K_2_O: 26-12-12, 525 kg/ha^-1^) | Uniform irrigation according to soil conditions |

**Note: AA, alfalfa monoculture; A/M, alfafa and maize intercropping.**

**Table S2 Topological properties of the diazotrophic molecular ecological networks in the AA and A/M systems across different sites**

| Network properties | Poor soil site | |  | Average soil site | |
| --- | --- | --- | --- | --- | --- |
|  | AA | A/M |  | AA | A/M |
| Total nodes | 145 | 116 |  | 78 | 84 |
| Total links (Negative links) | 1386 (614) | 581 (283) |  | 348 (159) | 391 (158) |
| Average degree (avgK) | 19.12 | 10.02 |  | 8.92 | 9.31 |
| Average clustering coefficient (avgCC) | 0.64 | 0.56 |  | 0.63 | 0.59 |
| Average path distance (GD) | 2.92 | 3.74 |  | 3.64 | 3.27 |
| Modularity (M) | 0.39 | 0.58 |  | 0.50 | 0.48 |


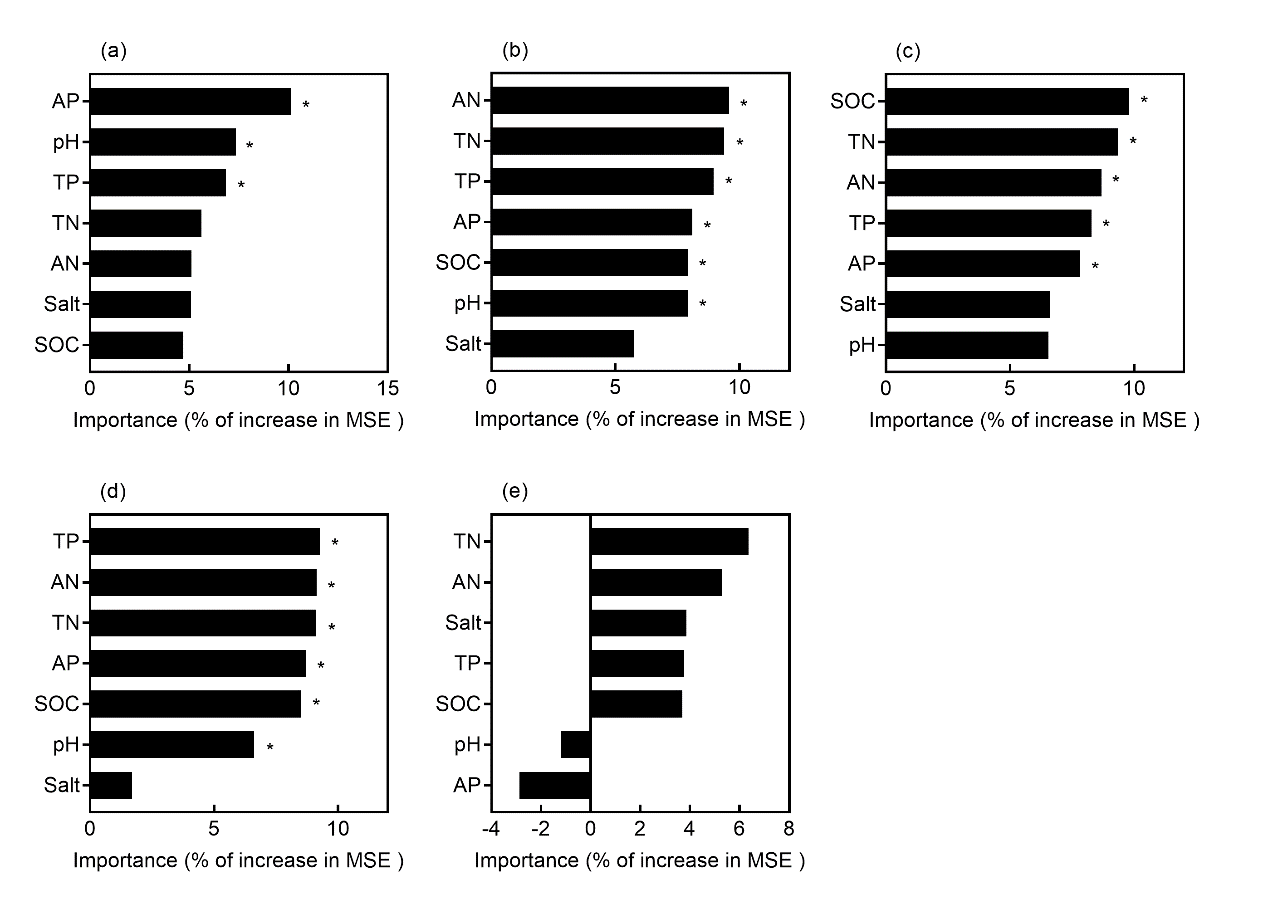
**Fig. S3 Random forest (RF) predictor importance (percentage of increase of mean square error, MSE) of soil properties as drivers for diazotrophic community diversity (a, richness; b, diversity; c, evenness) and structure (d, NMDS1; e, NMDS2) across study sites.**


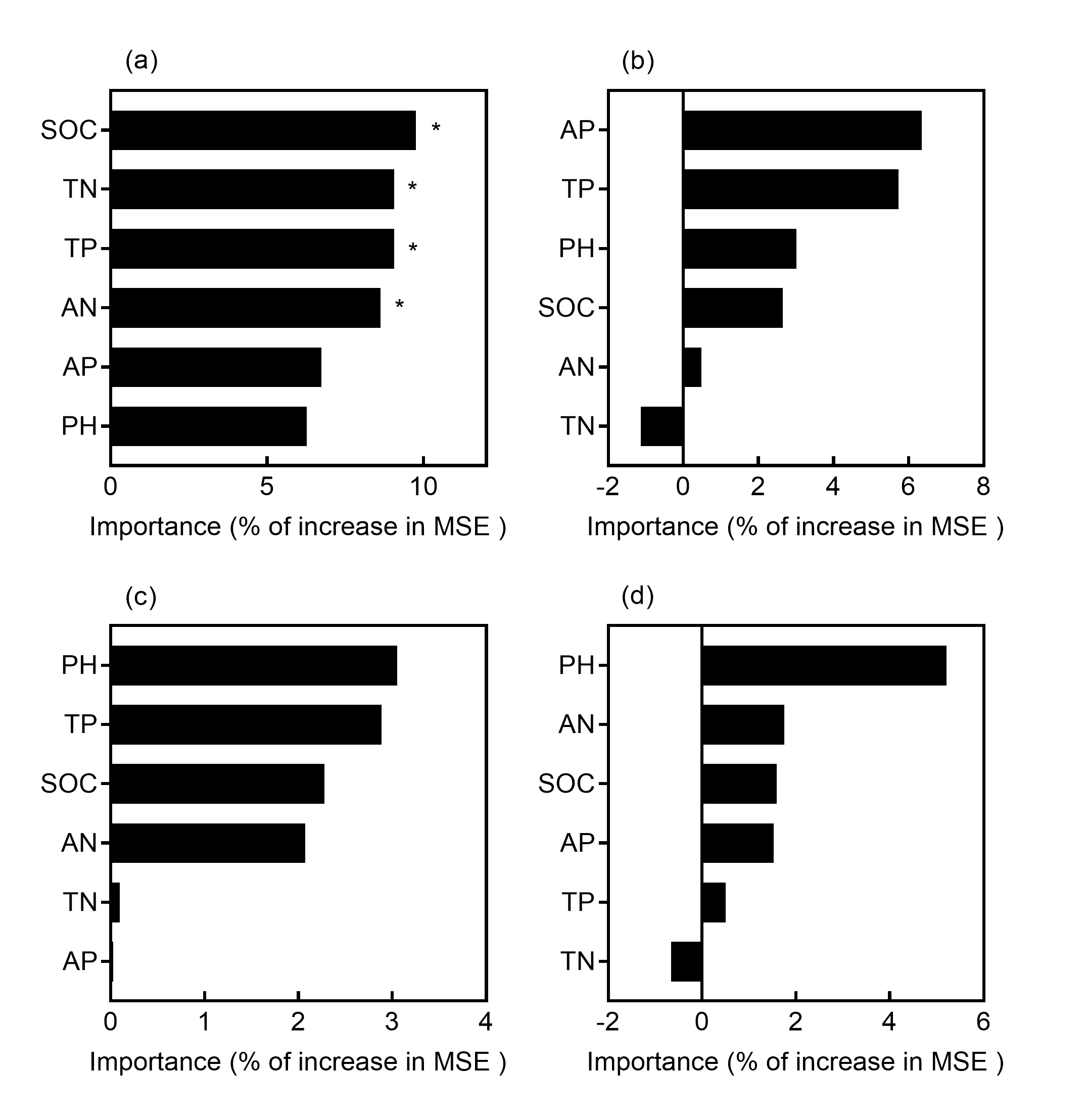


**Fig. S4 Random forest (RF) predictor importance (percentage of increase of mean square error, MSE) of soil properties as drivers for diazotrophic network structure attributes (a, nodes; b, degree; c, average path length; d, betweenness centralization) across study sites.**
